# Neuronal morphology as an instrument for information coding: studying the influence of axonal radius and branching points

**DOI:** 10.1186/1471-2202-14-S1-P357

**Published:** 2013-07-08

**Authors:** Netanel Ofer, Orit Shefi

**Affiliations:** 1 Faculty of Engineering, Bar Ilan University, Ramat Gan, 52900, Israel; 2 Bar Ilan Institute of Nanotechnologies and Advanced Materials, Bar Ilan University, Ramat Gan, 52900, Israel

## 

The cell's electrical behavior is affected from its morphology. The geometry could filter the spike repetition and consequently has a role in the information processing of the cell [1,2]. We examine the response of axons to different current stimuli using the Hodgkin Huxley cable model. We explore the influence of the cell morphology on the electrical activity pattern. We analyze the electrical response of axons with different morphology to different current stimuli using nonlinear dynamics tools. The electrical response patterns are presented in phase diagrams, bifurcation diagrams and 2D response diagrams.

**Figure 1:**
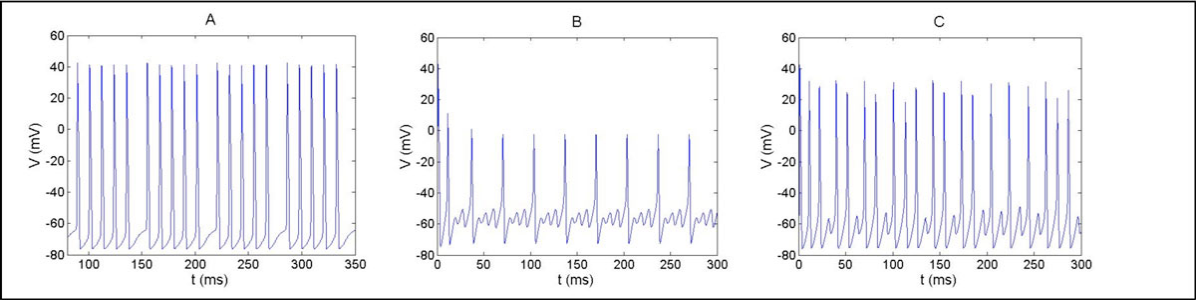
**A. Series of five spikes before failure for a current stimulus of 1.025 mA/cm^2 ^along the axon, 20µm radius**. **B**. Single spike followed by three failures for a current stimulus of 8.5 mA/cm^2 ^along the axon, 500µm radius. **C**. A chaotic response to a current stimulus of 4.064 mA/cm^2 ^along the axon, 238µm radius.

Normally a wide range of constant current stimuli, applied to one end of a simple cylindrical axon, leads to the generation of a train of action potentials propagating along the axon. We find that for a narrow range of high current stimuli, the stimulus leads to different patterns of response. These patterns include series of spikes followed by a single failure and a single spike followed by some failures. For specific current stimulus regimes, chaotic behavior is observed. Figure 1. represents the three responses along the axon for different radii of axon. A constant current stimulus of 1.025 mA/cm^2 ^injected to a 20µm axon radius results in the generation of five action potentials followed by a single failure (A). Current stimulus of 8.5 mA/cm^2 ^injected to a 500µm axon radius results in generation of a single action potential followed by three failures (B). Current stimulus of 4.064 mA/cm^2 ^injected to a 238µm axon radius leads to a chaotic response (C). Interestingly, similar behaviors can be generated along axonal trees at the branching points. A train of action potentials that propagates in the main branch bifurcates into daughter branches. Some of the spikes may fail to pass the junction point, leading to electrical response pattern of failures between spikes. This response pattern depends on the radii ratio between the branches of the mother and the daughters. Meaning, the geometry of the junction affects the electrical activity of the daughter axon. These results highly depend on the temperature.

Our results emphasize the critical role of the axonal morphology in neuronal electrical activity. These phenomena illustrate ways in which the pattern of activity may be controlled, demonstrating the ability to regulate the number of action potentials before failures, and suggest this behavior as instrumental in information coding.
